# COVID-Transformer: Interpretable COVID-19 Detection Using Vision Transformer for Healthcare

**DOI:** 10.3390/ijerph182111086

**Published:** 2021-10-21

**Authors:** Debaditya Shome, T. Kar, Sachi Nandan Mohanty, Prayag Tiwari, Khan Muhammad, Abdullah AlTameem, Yazhou Zhang, Abdul Khader Jilani Saudagar

**Affiliations:** 1 School of Electronics Engineering, KIIT Deemed to be University, Odisha 751024, India; 1804372@kiit.ac.in (D.S.); tkarfet@kiit.ac.in (T.K.); 2 Department of Computer Science & Engineering, Vardhaman College of Engineering (Autonomous), Hyderabad 501218, India; sachinandan09@gmail.com; 3 Department of Computer Science, Aalto University, 02150 Espoo, Finland; prayag.tiwari@aalto.fi; 4 Visual Analytics for Knowledge Laboratory (VIS2KNOW Lab), School of Convergence, College of Computing and Informatics, Sungkyunkwan University, Seoul 03063, Korea; 5 Information Systems Department, College of Computer and Information Sciences, Imam Mohammad Ibn Saud Islamic University (IMSIU), Riyadh 11432, Saudi Arabia; altameem@imamu.edu.sa; 6 Software Engineering College, Zhengzhou University of Light Industry, Zhengzhou 450001, China; yzzhang@zzuli.edu.cn

**Keywords:** vision transformer, COVID-19, deep learning, data science, healthcare, interpretability, transfer learning, grad-CAM

## Abstract

In the recent pandemic, accurate and rapid testing of patients remained a critical task in the diagnosis and control of COVID-19 disease spread in the healthcare industry. Because of the sudden increase in cases, most countries have faced scarcity and a low rate of testing. Chest X-rays have been shown in the literature to be a potential source of testing for COVID-19 patients, but manually checking X-ray reports is time-consuming and error-prone. Considering these limitations and the advancements in data science, we proposed a Vision Transformer-based deep learning pipeline for COVID-19 detection from chest X-ray-based imaging. Due to the lack of large data sets, we collected data from three open-source data sets of chest X-ray images and aggregated them to form a 30 K image data set, which is the largest publicly available collection of chest X-ray images in this domain to our knowledge. Our proposed transformer model effectively differentiates COVID-19 from normal chest X-rays with an accuracy of 98% along with an AUC score of 99% in the binary classification task. It distinguishes COVID-19, normal, and pneumonia patient’s X-rays with an accuracy of 92% and AUC score of 98% in the Multi-class classification task. For evaluation on our data set, we fine-tuned some of the widely used models in literature, namely, EfficientNetB0, InceptionV3, Resnet50, MobileNetV3, Xception, and DenseNet-121, as baselines. Our proposed transformer model outperformed them in terms of all metrics. In addition, a Grad-CAM based visualization is created which makes our approach interpretable by radiologists and can be used to monitor the progression of the disease in the affected lungs, assisting healthcare.

## 1. Introduction

As of June 2021, there have been 173 million COVID-19 cases worldwide, with new cases rapidly increasing at an alarming rate and showing no signs of abating [1]. If COVID-19 infection is not detected early enough, it can induce a flu-like sickness that can proceed to acute respiratory distress syndrome (ARDS), which can be deadly [2,3,4,5,6,7,8]. Due to limited resources and the amount of data accessible to the scientific community, early diagnosis of COVID-19 remains a tough challenge despite recent worldwide research efforts in healthcare [9]. RT-PCR has been the standard and approved diagnostic approach for COVID-19, however it has a number of drawbacks. It is costly, risky to medical staff, and there are just a few diagnostic test kits accessible. Medical imaging techniques such as X-ray and CT-based screening, on the other hand, are relatively safe, faster, and more widely available. X-ray imaging has been frequently utilized for COVID-19 screening in comparison to CT imaging as it takes less imaging time, is less expensive, and X-ray scanners are commonly available even in remote regions [10]. Because of the complicated morphological patterns of lung involvement, which can fluctuate in degree and appearance over time, the accuracy of a COVID-19 infection diagnosis using chest imaging is strongly reliant on radiological proficiency. The scarcity of competent radiologists, particularly in developing countries, affects the reliability of sophisticated chest examination interpretation. In a study by Cozzi et al. [11], it was found that chest X-ray imaging achieved a balanced real-world diagnostic performance with accuracy in the range of 76% to 86%, and 89% sensitivity. They showed that the specificity was much greater for experienced radiologists than that of less experienced ones. Deep Learning and Data Science are widely employed in many fields of medical imaging, and they have shown excellent results in Thoracic Imaging [12]. There have been many approaches for diagnosing COVID-19 from CT and X-ray images that made use of Deep Learning/Data Science recently.

There have been some efforts on using unsupervised learning based approaches for this task. For instance, in [13], Mittal et al. developed an unsupervised learning-based technique for COVID-19 diagnosis from multiple modalities of chest imaging. They used a novel clustering based Gravitational Search algorithm for labeling the images into covid and non-covid. They achieved an accuracy of 99.36% on the ultrasound dataset but their approach achieved only 64.41% on CT dataset. In [14], Rui et al. used a pulmonary opacity detecting model trained using unsupervised learning over a small dataset of COVID CT scan images and they achieved an accuracy of 95.5% for detection of COVID-19.

A few object detection-based approaches have also been used for detecting COVID-19. For example, the authors of [15] proposed a YOLO-based object detection model for detecting and differentiating COVID-19 from other thoracic diseases. Their model achieved a detection accuracy of 90.67%. In [16], Fatima et al. used single shot multi-box detector based object detection technique which creates bounding boxes over the areas of the chest X-rays and each bounding box is classified as normal or COVID-19. They report an accuracy of 92% for classifying COVID-19.

The most used approach for solving this task has been using deep convolutional neural networks (CNNs) with supervised learning [17,18,19]. As an example, a work by Mukherjee et al. [20] proposed a deep CNN-based architecture and trained it on a combined data set of chest X-ray and CT images where they achieved an overall accuracy of 96.28%. In [21], Li et al. proposed a stacked autoencoder model where the first four layers consist of four autoencoders to extract better features from CT images. The final model is built by chaining together these four autoencoders and linking them to the dense layer and the softmax classifier. The authors report achieving an accuracy of 94.7% on the CT images data set. In [22], Chakraborty et al. proposed Corona-Nidaan, a lightweight deep CNN architecture trained on chest X-ray data set with three classes which achieved an accuracy of 95%. In [23], the authors used transfer learning with a VGG-16 model pre-trained on pneumonia detection and further fine-tuned it over a COVID-19 detection data set achieving an accuracy of 93%, although they sometimes misclassify COVID-19, viral pneumonia, and normal cases. In [24], Khan et al. presented a Xception network-based architecture with pre-trained weights from ImageNet, which they fine tuned over a 1.2 K images data set, and they report an overall accuracy of 89.5% on multi-class classification. In [25], the authors proposed a two-level pipeline with an image segmentation block made up of a fully connected DenseNet backbone and a classification block where Resnet-18 was used patch-wise which achieved an accuracy of 91% upon training on a 354 images data set and upon evaluating over 99 images. In [26], Mishra et al. presented an approach with a two neural network-based system and reported a maximum accuracy of 96%. The authors of [27] used an attention based pre-trained VGG-16 model and fine-tuned it over three data sets with 1125, 1638, and 2138 images, respectively. Upon evaluation, they achieved an accuracy of 79.58%, 85.43%, 87.49% over the three data sets, respectively. Shankar et al. [28] proposed a BMO-CRNN algorithm for the covid-19 detection efficiently. In [29], Xueyu et al. fine-tuned multiple pre-trained models over a 2500 CT scan images data set and achieved 82.5% accuracy. Luz et al. [30] proposed models based on the EfficientNet family with a hierarchical classifier and achieved an overall accuracy of 93.9%. Pham et al. [31] presented a comprehensive study on transfer learning for COVID-19 detection from CT images by training and comparing 16 pre-trained models.

After reviewing the relevant literature, it is clear that despite the effectiveness of deep learning-based frameworks in COVID-19 identification, there are a few flaws. Most of the models have been trained and evaluated on data sets with few samples which can lead to improper generalization due to which the model might perform very poorly in real world and having a small test set might result in missing out on false positives or negatives. With this motivation, we have conducted this research with main contributions highlighted as follows:

- Due to lack of large public data sets, we collected and merged three standard data sets (https://data.mendeley.com/datasets/9xkhgts2s6 (accessed on 1 May 2021)), (https://data.mendeley.com/datasets/8h65ywd2jr/3 (accessed on 1 May 2021)), (https://www.kaggle.com/endiqq/largest-covid19-dataset) (accessed on 1 May 2021) to form a 30 K chest X-ray images COVID-19 data set for multi-class classification and a 20 K images data set for binary classification. These two data sets have equal number of images in each class making it the largest and balanced data set on COVID-19 imaging-based detection available as open-source, which can help the research community in training much more accurate and generalizable models in the future.
- We implemented a model based on Vision Transformer (ViT) architecture on both data sets and achieved a state-of-the-art overall accuracy of 98.4% in distinguishing COVID-19 positive from normal X-rays, and an accuracy of 92.4% in distinguishing COVID-19 from pneumonia and normal X-ray images.
- For evaluation, we fine-tuned multiple state-of-the-art baseline models which are widely used in literature such as Inception-V3, Resnet-V2, EfficientNet-B0, MobileNet-V2, VGG-16, Xception, and DenseNet-121 on both of the data sets and compared these with our proposed model on multiple standard metrics.
- For better model interpretability and ease of diagnosis, we created Grad-CAM-based visualizations of COVID-19 progression in the lungs, which assists the diagnosis process for healthcare.

The rest of this paper is divided into four sections, with multiple subsections within each of them. Section 2 is focused on the proposed model’s architecture and training pipeline. Section 3 discusses the fine-grained details of our data collection and preprocessing pipeline, followed by performance evaluation, comparison with baselines, and interpretable visualizations. Section 4 presents an overview on the real-world utility of our methodology in order to assist health services during emergency times. Section 5 concludes this work.

## 2. Model Architecture and Pipeline

This section covers information about the proposed transfer learning model as well as critical parameter evaluations, fine-tuning steps, and model comparisons.

### 2.1. Architecture

**Figure 1:**
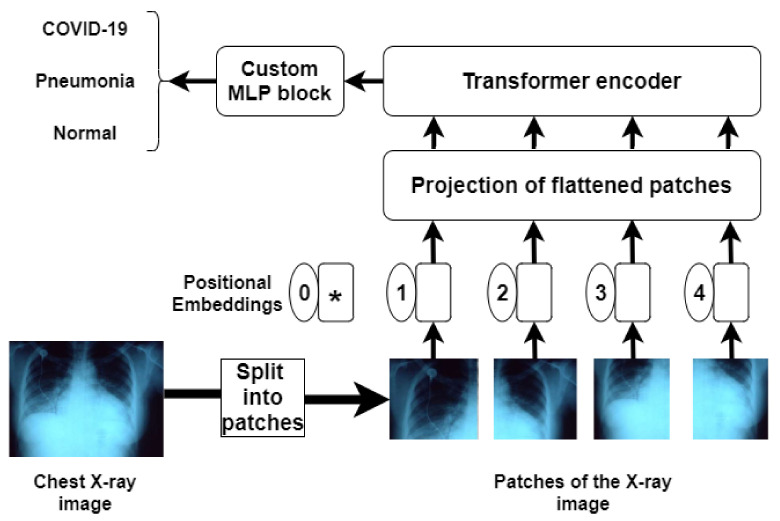
The proposed ViT model for COVID-19 detection.

After the success of Transformers in solving natural language processing problems [32], Dosovitskiy et al. in [33] presented the Vision Transformer (ViT) model. When trained on enough data, ViT beats state-of-the-art CNN with approximately four times less computing resources. ViT tries to resemble the original transformer architecture [34] as much as possible. We designed a COVID-19 detection pipeline utilizing the Vision Transformer model and fine-tuned it on our dataset with a custom MLP block. The initial part of the network has a Patch Encoder layer which reshapes the input image into multiple flattened patches. Along with the patches, positional embeddings are added to form a sequence, because only sequential data are compatible with the Transformer encoders. The Transformer encoder used is the same as that in [34] and contains multi-headed self-attention layers and multiple Multi-layer Perceptron (MLP) blocks. ViT’s self-attention layer enables it to integrate information globally throughout the full picture. To recreate the visual structure from the training data, ViT learns to encode the relative placement of the patches. Self-attention has a quadratic cost as each pixel in the image is given as input, self-attention requires each pixel to pay attention to every other pixel. Because the quadratic cost of self-attention is prohibitively expensive and does not scale to a reasonable input size, the image is separated into patches. Because it does not establish any additional dependencies between the training images, Layer Norm is used before each block which assists in reducing training time and improving generalization performance. The overall architecture has been illustrated in Figure 1.

### 2.2. Fine-Tuning Procedure

**Figure 2:**
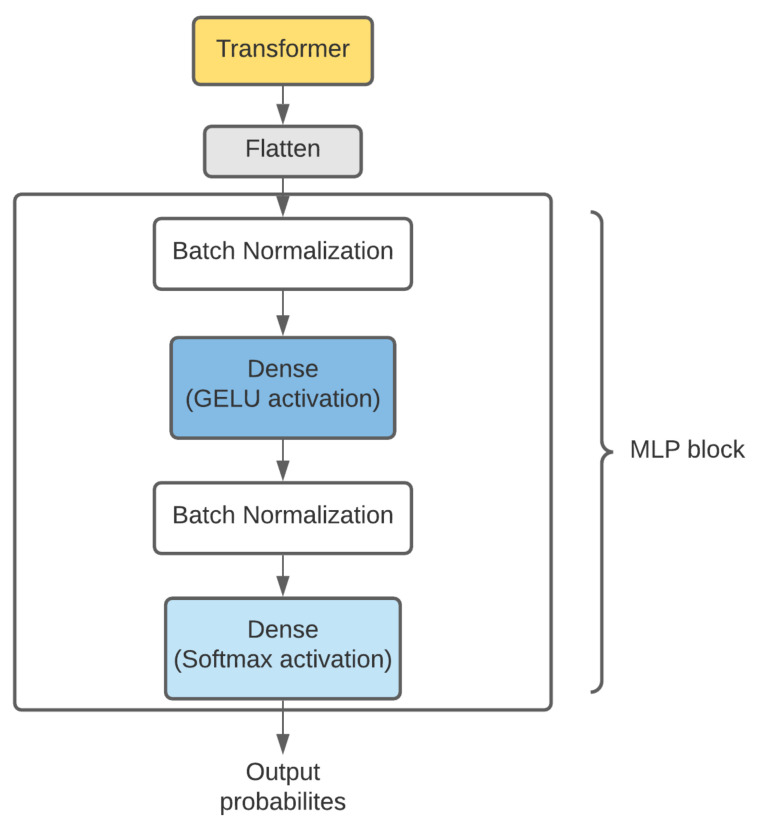
Custom MLP block attached to the Vision Transformer.

We used the ViT L-16 model for the initial stage of our model, which is the “Large” variant with a patch size of 16 × 16. The ViT model had pre-trained weights from training on ImageNet data [35]. This initial stage consists of 23 transformer encoder layers stacked on top of each other. We removed the pre-trained MLP prediction block and attached an untrained set of feed-forward layers constituting the custom MLP block which can be seen in Figure 2. The flattened output of the final transformer encoder is passed through two sets of batch normalization and dense layers constituting the MLP block. Batch normalization is a neural network layer that allows the model’s other layers to learn more independently [36]. It is used to make the output of the preceding layers more natural and to make the activations scale the input layer. Learning becomes more efficient when batch normalization is utilized, and it may also be employed as a regularization to prevent model overfitting. The first dense layer consists of a Gaussian error linear unit (GELU)-based activation with 120 neurons. GELU has been widely used in revolutionary transformer models such as GPT-2 [37], BERT [38], and also in vision transformers [33] due to its deterministic nonlinearity that encapsulates a stochastic regularization effect [39], which leads to a major performance boost in most models with complex transformer architectures. The last dense layer has softmax activation, and we use L2 regularization [40] to minimize overfitting as much as possible.

### 2.3. Model Training Mechanism

We use the NovoGrad optimizer with a categorical cross-entropy loss function to train our model for multi-class classification, and binary cross-entropy loss in the case of binary classification. In each case, label smoothing of 0.3 was added which helps to make the neural network generalize on unseen data by adding noise to the labels [41]. NovoGrad performs similarly to SGD but with gradient normalization per layer, making the optimizer more robust to initial learning rate selection. When compared to Adam, NovoGrad uses less memory and is more numerically stable due to which the training time of our model reduced without a drop in performance. It broadens Adam and decouples weight decay from regularization. It also has half the memory cost of Adam and similar memory needs to SGD with momentum. We also use the adaptive learning rate scheduler and callbacks from the Keras library [42] which automatically reduces the learning rate and stops overtraining the model if the validation accuracy does not improve. The data set was randomly split into train/validation/test sets with 75%/15%/10% of all the images, respectively.

**Figure 3:**
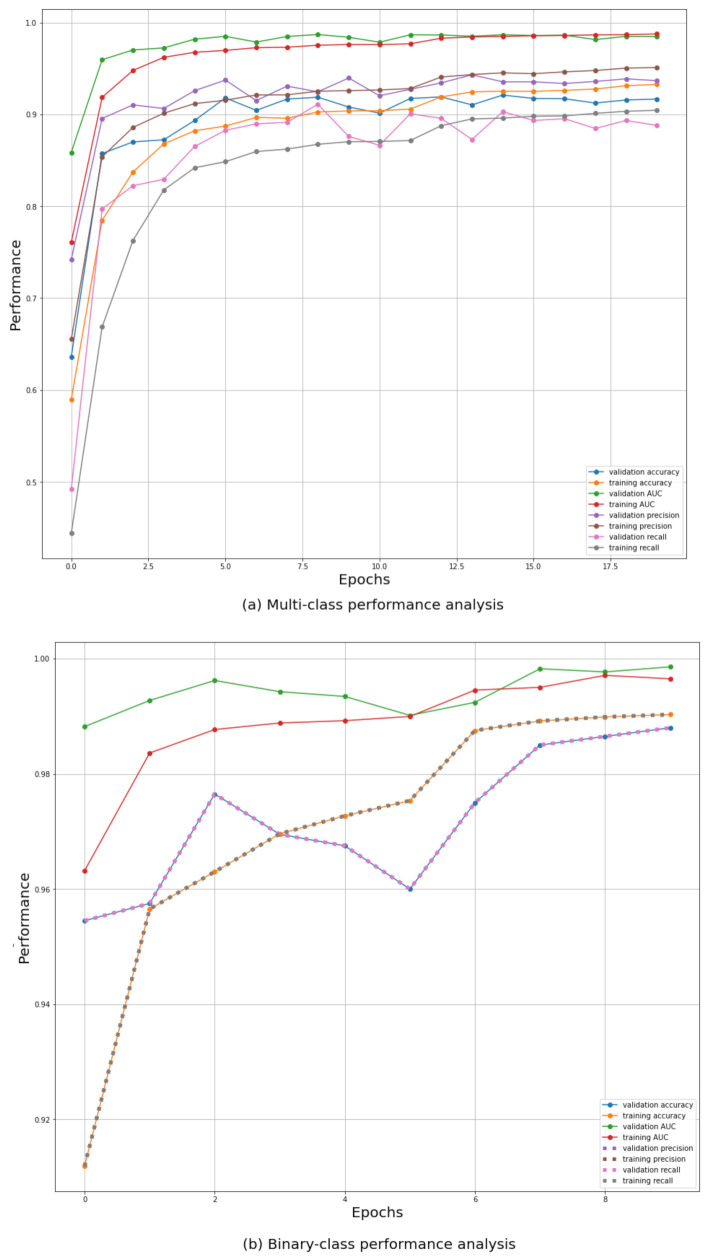
Performance measures during training.

As seen in Figure 3, multiple metrics were monitored during the training process where all of those showed a progressively increasing curve even on validation.

1 **Accuracy:** The most common performance metric in any classification problem is the accuracy metric. For the multi-class classification, the categorical accuracy was chosen which resembles the average accuracy over all the three classes of chest X-ray images. The binary classification involved the binary accuracy metric which measures how many times the predicted label matches the true label for the chest X-ray image.
2 **AUC score:** The area under the ROC curve (AUC) score shows how well predictions are ranked across all the classes and how much the model can distinguish between each class. It ensures that performance across all feasible categorization criteria is aggregated. It has been proved in the literature that AUC score is a more robust metric to measure the ability of a classifier than the accuracy [43].
3 **Precision:** Precision is defined as the number of true positives divided by the number of true positives plus the number of false positives.
4 **Recall:** Recall is defined as the number of true positives divided by the number of true positives plus the number of false negatives.

## 3. Experimental Results and Discussion

### 3.1. Data Set

**Figure 4:**
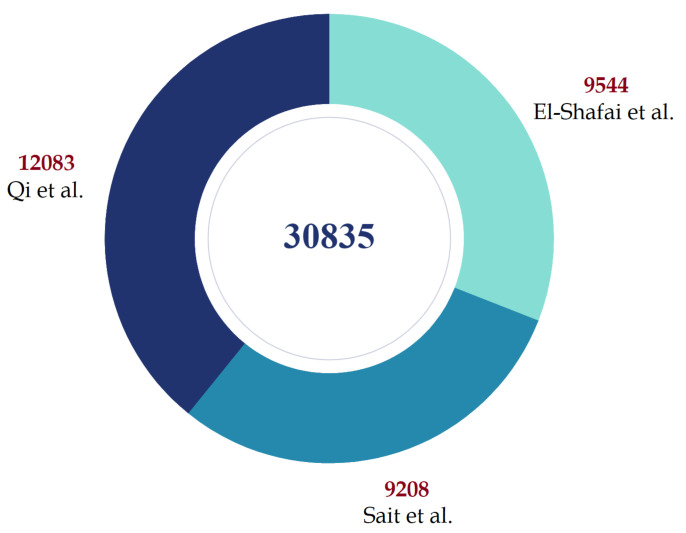
Data set distribution and description.

We constructed a three-class data set of 30 K chest X-ray pictures with labels COVID-19—for patients with COVID-19 infection, normal—for stable patients, and pneumonia—for patients with viral and bacterial pneumonia, following the pattern of likely classes reported in the literature. We took 5500 COVID images and 4044 normal images from El-Shafai et al. [44]. Another 1281 COVID-19 images, 3270 normal images, and 4657 pneumonia images were taken from Sait et al. [45]. Finally, we took 3000 normal images, 6045 pneumonia images, and 4038 COVID-19 images from the COVID-Ti data set by Qi et al. [46]. The distribution of the aggregated data has been visually illustrated in Figure 4.

To make our data set completely balanced, we sampled top 10 K images from each class by ranking them based on resolution, making it a chest X-ray data set of 30 K images for COVID-19 detection. This is, to the best of our knowledge, the largest open source collection of chest X-ray images for the detection of COVID-19 and pneumonia till date.

### 3.2. Preprocessing

Each image in the gathered data set is passed through a minimal image pre-processing pipeline which ensures to make all images compatible for the model training. The following are the steps in the pipeline:

1 **Resize:** As neural network models have a fixed-size input layer, all images must be scaled to the same size. Therefore, we resize all the images in the data set to 224 × 224 pixels.
2 **Interpolation:** There are a few images in the data set which are of size lesser than 224 × 224. While increasing their size, the estimation of new pixels needs to be done efficiently in order to retain quality. This process is termed “Interpolation” of images. For our pipeline we used the nearest neighbor interpolation, in which the closest pixel value to the supplied input coordinates is used to approximate the output pixel value. This approach is straightforward to implement, and there is no bogus data in the end result [47].

### 3.3. Data Augmentation

In order to develop accurate and generalizable deep learning models, supervised learning requires large amounts of data. In our training pipeline, we employed a variety of data augmentation techniques such as random rotation, width shift, height shifts, and flipping which have been demonstrated in the literature to be beneficial in increasing deep learning model performance [48,49].

### 3.4. Testing Environment

All the training and testing pipelines for the proposed models, as well as baselines were implemented using TensorFlow 2.4 framework [50] in a Python 3.8 virtual environment. The graphics processing unit used in the training pipeline was a 4.1 TFLOPS Tesla K80. The net available RAM was 24 GB. Jupyter notebooks were utilized to conduct the experiments.

### 3.5. Model Evaluation

**Figure 5:**
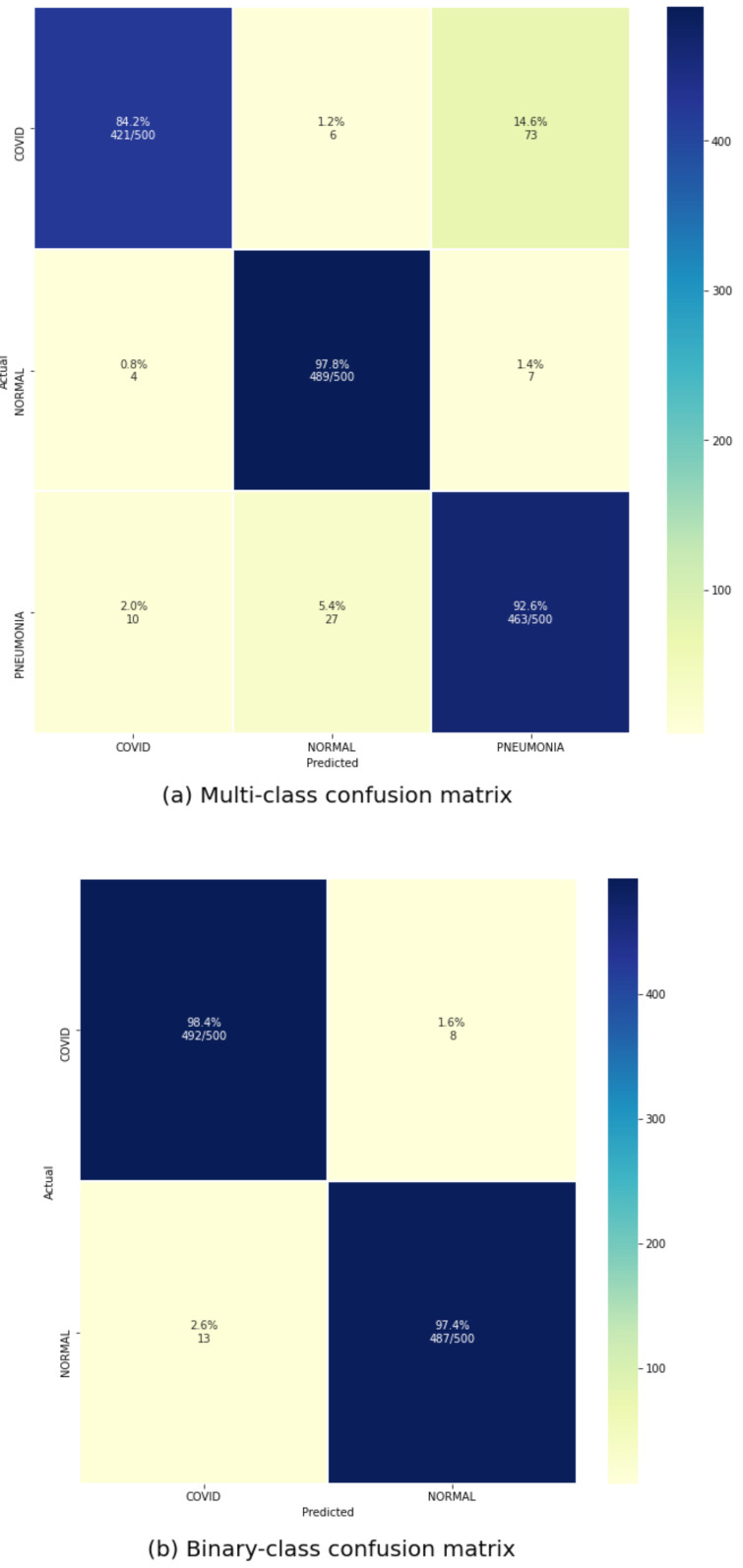
Confusion matrix for both types of classification.

Our proposed COVID-Transformer was evaluated over the test set of both of our multi-class and binary classification data sets. It can be observed from Figure 5a that our model is capable of distinguishing between all the three classes very accurately. Although the number of false positives and true negatives is lower, the model sometimes confuses between COVID-19 and other types of pneumonia, which is acceptable as COVID-19 is itself a form of pneumonia and it is very tough even for expert radiologists to distinguish between the two. As observed in Figure 5b, our proposed model performs extremely well over the binary classification data set with only 21 out of 1000 images misclassified. The overall performance metrics over the test data sets have been outlined in Table 1. The multi-class classification model works well, with an accuracy of 92% and an AUC score of 98%. In this situation, the accuracy is lower than the AUC score because only images projected as pneumonia but really COVID-19 are misclassified, while all other categories are correctly classified, hence the AUC score is not much affected. The binary classification model achieves an accuracy of 98% and an AUC score of 99% which is suitable for real-world deployment as a diagnosis tool for detecting COVID-19 as it has significantly higher performance than standard RT-PCR tests.

### 3.6. Ablation Experiments

In order to ensure that our transfer learning architecture is optimal, we conduct a comprehensive ablation study on the multi-class classification data set. We first experiment by modifying the custom block using different number of layers, activations, and order of layers. First, we observe that using only one dense layer with Batch normalization and ReLU activation, the accuracy drops down to 90%. Upon removing the Batch normalization the accuracy further degrades to 89%. However, if we replace ReLU with the GeLU activation function, a single dense layer with Batch normalization achieves an accuracy of 91% and 90% without Batch normalization. This shows that GeLU activation is slightly more effective in processing the outputs of the stacked transformer encoders compared to the ReLU activation. Next, we further experiment with a custom MLP block of two GeLU activated dense layers with and without batch normalization, where the accuracy increases to 92% and 91%, respectively. However, if we further add another dense layer with and without batch normalization, the training accuracy increases whereas the testing accuracy drops to 88% and 89%, respectively. This is a clear indication that our model results in overfitting beyond 2 dense layers, thus we decide to keep a custom MLP block with two batch normalization and dense layers in the final architecture.

### 3.7. Comparison with Baseline Models

As our data set has not been evaluated using other models in the literature, we fine-tuned some of the widely used state-of-the-art models on both variants of our data set. All the data preparation and image augmentation steps are same for the baselines, except for some of the preprocessing functions which are necessary for input to the models. For Inception-V3 and Xception fine-tuning, the images were re-sized to 299 × 299 pixels. The same data augmentation techniques as for our proposed COVID-Transformer model were used. The MobileNet-V2, ResNet-V2-50, DenseNet-121, VGG-16, and EfficientNet-B0 models have a standard size requirement of 224 × 224 pixels, which is same as of our COVID-Transformer model, thus we used the same data preprocessing and augmentation steps for fine-tuning them. From Table 2, it can be noted that among the baselines MobileNet-V3 and Xception perform the best with 90% Accuracy on the multi-class classification problem. Our COVID-Transformer model outperforms all the baselines in terms of accuracy, precision, F1 score, and AUC score.

### 3.8. Grad-Cam Visualization

**Figure 6:**
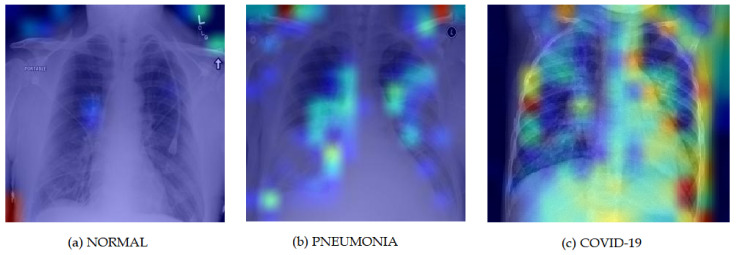
Grad-CAM visualization for the three classes.

For better visual representation and model interpretability, the Grad CAM Map-based illustration introduced by Selvaraju et al. [51] is shown in Figure 6. The Grad CAM Map visualization has the capability to highlight affected areas of the lungs that are significant for disease predictions as well as disease development. The images are obtained by passing the output of the embedding layer present in our model at the beginning just after the input layer.

Figure 6a shows a normal patient with no disease having no highlighted region in the lungs. Figure 6b shows pneumonia patient’s lungs with affected regions highlighted in blue and green. Figure 6c shows a COVID-19 infected patient with mostly yellow and red highlighted regions which indicate severe infection. The figure clearly shows that our suggested methodology recognizes and differentiates relevant impacted areas from COVID-19 and other pneumonia images. COVID-19 impacts the lungs considerably more intensively than other types of pneumonia, thus our model emphasizes this by highlighting yellow and red areas in the COVID patient’s X-ray image.

## 4. Case Study in Medical Services

**Figure 7:**
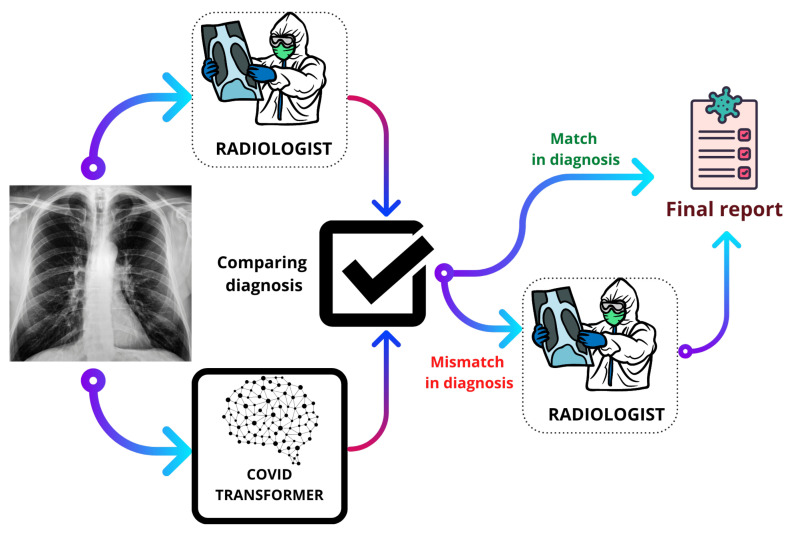
Flow of deployable solution.

Health systems in both rich and poor nations were overburdened by the COVID-19 outbreak. Sustainable Development Goals (SDGs) planned for 2025 will be affected by the pandemic-related losses, there is no question about it. As a result of the epidemic, there was a window of opportunity to take use of current digital solutions and discover new ones. These solutions can aid in the fulfillment of the SDGs, particularly those that pertain to health. In this sense, achieving global health coverage is an important SDG. Early diagnosis is an important factor in reducing the number of deaths from COVID-19, which almost becomes impossible when there is a steep rise in infections concentrated in a particular location. If an infected individual is isolated at the right time, multiple infections from further transmissions can be prevented. Our proposed method for X-ray based detection of COVID-19 would be an efficient addition to the healthcare system boosting the global health coverage. It can be used as an aid for radiologists to reduce human errors in their diagnosis, as well as can be used as a single tool to detect COVID-19 in places where radiologists are not adequate due to infections rising at a breakneck pace. Figure 7 shows the typical flow of our method when deployed in a real-world setting to have zero human error diagnosis.

## 5. Conclusions

In this research, we proposed a robust and interpretable deep learning model that can efficiently diagnose COVID-19 infection at scale in real-world situations for healthcare. For this objective, a 30 K chest X-ray image collection was produced by combining several open-source data sets. The model architecture chosen was based on the Vision Transformer and it showed high performance with accuracy and AUC score as high as 98% and 99%, respectively. For making our model trustworthy, we made an interpretable inference pipeline with Grad-CAM based visualizations per image. We believe that with the help of our proposed approach, chest X-ray images can also be used as a crude and low-cost bedside diagnostic tool for detecting COVID-19. This may be extremely valuable in areas where quick testing is unavailable, and it may also be used as a second screening method after the standard RT-PCR test to verify that any true negative or false positive cases do not occur. Our future work will focus on proposing another variant of the Vision Transformer for further improving the performance, given the availability of larger data sets.

## Figures and Tables

**Table 1:** Evaluation of the proposed model.

Model	Accuracy	Precision	Recall	F1 Score	AUC
**Binary-class**	0.98	0.97	0.97	0.97	0.99
**Multi-class**	0.92	0.93	0.89	0.91	0.98

**Table 2:** Performance comparison of our COVID-Transformer with baseline models on the multi-class classification problem.

Model	Accuracy	Precision	Recall	F1 Score	AUC
**Inception-V3** [31]	0.90	0.89	0.91	0.89	0.92
**EfficientNet-B0** [30]	0.89	0.88	0.89	0.88	0.92
**MobileNet-V2** [31]	0.90	0.90	0.89	0.90	0.92
**ResNet-V2** [29,31]	0.88	0.87	0.86	0.86	0.93
**VGG-16** [23,27,29,31]	0.87	0.87	0.85	0.86	0.90
**Xception** [24,31]	0.90	0.92	0.87	0.90	0.93
**DenseNet-121** [25,29,31]	0.88	0.90	0.85	0.87	0.92
**COVID-Transformer (Ours)**	**0.92**	**0.93**	**0.89**	**0.91**	**0.98**

## Data Availability

https://github.com/DebadityaShome/COVID-Transformer.

## References

[1] World-Health-Organization COVID-19 Weekly Epidemiological Update. https://www.who.int/.

[2] Lang T. (2020). Plug COVID-19 research gaps in detection, prevention and care. Nature.

[3] Yang P., Wang X. (2020). COVID-19: A new challenge for human beings. Cell. Mol. Immunol..

[4] Laajaj R., De Los Rios C., Sarmiento-Barbieri I., Aristizabal D., Behrentz E., Bernal R., Buitrago G., Cucunubá Z., de la Hoz F., Gaviria A. (2021). COVID-19 spread, detection, and dynamics in Bogota, Colombia. Nat. Commun..

[5] Vepa A., Saleem A., Rakhshan K., Daneshkhah A., Sedighi T., Shohaimi S., Omar A., Salari N., Chatrabgoun O., Dharmaraj D. (2021). Using Machine Learning Algorithms to Develop a Clinical Decision-Making Tool for COVID-19 Inpatients. Int. J. Environ. Res. Public Health.

[6] Ghibu S., Juncan A.M., Rus L.L., Frum A., Dobrea C.M., Chiş A.A., Gligor F.G., Morgovan C. (2021). The Particularities of Pharmaceutical Care in Improving Public Health Service During the COVID-19 Pandemic. Int. J. Environ. Res. Public Health.

[7] Xu T. (2021). Psychological Distress of International Students during the COVID-19 Pandemic in China: Multidimensional Effects of External Environment, Individuals’ Behavior, and Their Values. Int. J. Environ. Res. Public Health.

[8] Cass A.L., Slining M.M., Carson C., Cassidy J., Epright M.C., Gilchrist A.E., Peterson K., Wheeler J.F. (2021). Risk Management of COVID-19 in the Residential Educational Setting: Lessons Learned and Implications for Moving Forward. Int. J. Environ. Res. Public Health.

[9] Ting D.S.W., Carin L., Dzau V., Wong T.Y. (2020). Digital technology and COVID-19. Nat. Med..

[10] Nayak S.R., Nayak D.R., Sinha U., Arora V., Pachori R.B. (2021). Application of deep learning techniques for detection of COVID-19 cases using chest X-ray images: A comprehensive study. Biomed. Signal Process. Control.

[11] Cozzi A., Schiaffino S., Arpaia F., Della Pepa G., Tritella S., Bertolotti P., Menicagli L., Monaco C.G., Carbonaro L.A., Spairani R. (2020). Chest X-ray in the COVID-19 pandemic: Radiologists’ real-world reader performance. Eur. J. Radiol..

[12] Zhou S.K., Greenspan H., Davatzikos C., Duncan J.S., Van Ginneken B., Madabhushi A., Prince J.L., Rueckert D., Summers R.M. (2021). A Review of Deep Learning in Medical Imaging: Imaging Traits, Technology Trends, Case Studies With Progress Highlights, and Future Promises. Proc. IEEE.

[13] Mittal H., Pandey A.C., Pal R., Tripathi A. (2021). A new clustering method for the diagnosis of CoVID19 using medical images. Appl. Intell..

[14] Xu R., Cao X., Wang Y., Chen Y.W., Ye X., Lin L., Zhu W., Chen C., Xu F., Zhou Y. Unsupervised Detection of Pulmonary Opacities for Computer-Aided Diagnosis of COVID-19 on CT Images. Proceedings of the 2020 25th International Conference on Pattern Recognition (ICPR).

[15] Al-antari M.A., Hua C.H., Bang J., Lee S. (2021). Fast deep learning computer-aided diagnosis of COVID-19 based on digital chest X-ray images. Appl. Intell..

[16] Saiz F.A., Barandiaran I. (2020). COVID-19 detection in chest X-ray images using a deep learning approach. Int. J. Interact. Multimed. Artif. Intell..

[17] Aslan M.F., Unlersen M.F., Sabanci K., Durdu A. (2021). CNN-based transfer learning—BiLSTM network: A novel approach for COVID-19 infection detection. Appl. Soft Comput..

[18] Marques G., Agarwal D., de la Torre Díez I. (2020). Automated medical diagnosis of COVID-19 through EfficientNet convolutional neural network. Appl. Soft Comput..

[19] Demir F. (2021). DeepCoroNet: A deep LSTM approach for automated detection of COVID-19 cases from chest X-ray images. Appl. Soft Comput..

[20] Mukherjee H., Ghosh S., Dhar A., Obaidullah S.M., Santosh K., Roy K. (2020). Deep neural network to detect COVID-19: One architecture for both CT Scans and Chest X-rays. Appl. Intell..

[21] Li D., Fu Z., Xu J. (2020). Stacked-autoencoder-based model for COVID-19 diagnosis on CT images. Appl. Intell..

[22] Chakraborty M., Dhavale S.V., Ingole J. (2021). Corona-Nidaan: Lightweight deep convolutional neural network for chest X-ray based COVID-19 infection detection. Appl. Intell..

[23] Perumal V., Narayanan V., Rajasekar S.J.S. (2021). Detection of COVID-19 using CXR and CT images using Transfer Learning and Haralick features. Appl. Intell..

[24] Khan A.I., Shah J.L., Bhat M.M. (2020). CoroNet: A deep neural network for detection and diagnosis of COVID-19 from chest X-ray images. Comput. Methods Prog. Biomed..

[25] Oh Y., Park S., Ye J.C. (2020). Deep learning covid-19 features on cxr using limited training data sets. IEEE Trans. Med. Imaging.

[26] Mishra M., Parashar V., Shimpi R. Development and evaluation of an AI System for early detection of Covid-19 pneumonia using X-ray (Student Consortium). Proceedings of the 2020 IEEE Sixth International Conference on Multimedia Big Data (BigMM).

[27] Sitaula C., Hossain M.B. (2020). Attention-based VGG-16 model for COVID-19 chest X-ray image classification. Appl. Intell..

[28] Shankar K., Perumal E., Díaz V.G., Tiwari P., Gupta D., Saudagar A.K.J., Muhammad K. (2021). An optimal cascaded recurrent neural network for intelligent COVID-19 detection using Chest X-ray images. Appl. Soft Comput..

[29] Wu X., Wang Z., Hu S. Recognizing COVID-19 positive: Through CT images. Proceedings of the 2020 Chinese Automation Congress (CAC).

[30] Luz E., Silva P., Silva R., Silva L., Guimarães J., Miozzo G., Moreira G., Menotti D. (2021). Towards an effective and efficient deep learning model for COVID-19 patterns detection in X-ray images. Res. Biomed. Eng..

[31] Pham T.D. (2020). A comprehensive study on classification of COVID-19 on computed tomography with pretrained convolutional neural networks. Sci. Rep..

[32] Wang B., Xie Q., Pei J., Tiwari P., Li Z. (2021). Pre-trained Language Models in Biomedical Domain: A Survey from Multiscale Perspective. arXiv.

[33] Dosovitskiy A., Beyer L., Kolesnikov A., Weissenborn D., Zhai X., Unterthiner T., Dehghani M., Minderer M., Heigold G., Gelly S. (2020). An image is worth 16×16 words: Transformers for image recognition at scale. arXiv.

[34] Vaswani A., Shazeer N., Parmar N., Uszkoreit J., Jones L., Gomez A.N., Kaiser L., Polosukhin I. (2017). Attention is all you need. arXiv.

[35] Deng J., Dong W., Socher R., Li L.J., Li K., Fei-Fei L. Imagenet: A large-scale hierarchical image database. Proceedings of the 2009 IEEE Conference on Computer Vision and Pattern Recognition.

[36] Ioffe S., Szegedy C. (2015). Batch normalization: Accelerating deep network training by reducing internal covariate shift. International Conference on Machine Learning.

[37] Radford A., Wu J., Child R., Luan D., Amodei D., Sutskever I. (2019). Language models are unsupervised multitask learners. OpenAI Blog.

[38] Devlin J., Chang M.W., Lee K., Toutanova K. (2019). BERT: Pre-training of Deep Bidirectional Transformers for Language Understanding. https://arxiv.org/abs/1810.04805.

[39] Hendrycks D., Gimpel K. (2016). Gaussian error linear units (gelus). arXiv.

[40] Cortes C., Mohri M., Rostamizadeh A. (2012). L2 regularization for learning kernels. arXiv.

[41] Müller R., Kornblith S., Hinton G. (2019). When does label smoothing help?. arXiv.

[42] Chollet F. (2018). Keras: The python deep learning library. https://ui.adsabs.harvard.edu/abs/2018ascl.soft06022C/abstract.

[43] Ling C.X., Huang J., Zhang H. (2003). AUC: A statistically consistent and more discriminating measure than accuracy. Ijcai.

[44] El-Shafai W., Abd El-Samie F. (2020). Extensive COVID-19 X-ray and CT Chest Images Dataset. Mendeley Data.

[45] Sait U., Lal K.G., Prajapati S., Bhaumik R., Kumar T., Sanjana S., Bhalla K. (2020). Curated Dataset for COVID-19 Posterior-Anterior Chest Radiography Images (X-rays). Mendeley Data.

[46] Qi X., Brown L.G., Foran D.J., Nosher J., Hacihaliloglu I. (2021). Chest X-ray image phase features for improved diagnosis of COVID-19 using convolutional neural network. Int. J. Comput. Assist. Radiol. Surg..

[47] Devaraj S.J. (2019). Emerging Paradigms in Transform-Based Medical Image Compression for Telemedicine Environment. Telemedicine Technologies.

[48] Hussain Z., Gimenez F., Yi D., Rubin D. (2017). Differential data augmentation techniques for medical imaging classification tasks. AMIA Annual Symposium Proceedings.

[49] Muhammad K., Khan S., Del Ser J., de Albuquerque V.H.C. (2020). Deep learning for multigrade brain tumor classification in smart healthcare systems: A prospective survey. IEEE Trans. Neural Netw. Learn. Syst..

[50] Abadi M., Agarwal A., Barham P., Brevdo E., Chen Z., Citro C., Corrado G.S., Davis A., Dean J., Devin M. (2015). TensorFlow: Large-Scale Machine Learning on Heterogeneous Systems. https://www.tensorflow.org/.

[51] Selvaraju R.R., Cogswell M., Das A., Vedantam R., Parikh D., Batra D. Grad-cam: Visual explanations from deep networks via gradient-based localization. Proceedings of the IEEE International Conference on Computer Vision.

